# TRANEXAMIC ACID ACTION ON LIVER REGENERATION AFTER PARTIAL HEPATECTOMY: EXPERIMENTAL MODEL IN RATS

**DOI:** 10.1590/0102-6720201600020009

**Published:** 2016

**Authors:** Felipe Antonio SOBRAL, Henrique DAGA, Henrique Nogueira RASERA, Matheus da Rocha PINHEIRO, Igor Furlan CELLA, Igor Henrique MORAIS, Luciana de Oliveira MARQUES, Luiz Martins COLLAÇO

**Affiliations:** 1 Postgraduate Program in Principles of Surgery, Evangelic Faculty of Paraná/University Evangelic Hospital of Curitiba/Medical Research Institute; 2 Nossa Senhora das Graças Hospital, Curitiba, PR, Brazil.

**Keywords:** Tranexamic acid, Hepatectomy, Liver regeneration

## Abstract

**Background:**

Different lesions may affect the liver resulting in harmful stimuli. Some therapeutic procedures to treat those injuries depend on liver regeneration to increase functional capacity of this organ.

**Aim:**

Evaluate the effects of tranexamic acid on liver regeneration after partial hepatectomy in rats.

**Method:**

40 rats (*Rattus norvegicus albinus*, Rodentia mammalia) of Wistar-UP lineage were randomly divided into two groups named control (CT) and tranexamic acid (ATX), with 20 rats in each. Both groups were subdivided, according to liver regeneration time of 32 h or seven days after the rats had been operated. The organ regeneration was evaluated through weight and histology, stained with HE and PCNA.

**Results:**

The average animal weight of ATX and CT 7 days groups before surgery were 411.2 g and 432.7 g, and 371.3 g and 392.9 g after the regeneration time, respectively. The average number of mitotic cells stained with HE for the ATX and CT 7 days groups were 33.7 and 32.6 mitosis, and 14.5 and 14.9 for the ATX and CT 32 h groups, respectively. When stained with proliferating cell nuclear antigen, the numbers of mitotic cells counted were 849.7 for the ATX 7 days, 301.8 for the CT 7 days groups, 814.2 for the ATX 32 hand 848.1 for the CT 32 h groups.

**Conclusion:**

Tranexamic acid was effective in liver regeneration, but in longer period after partial hepatectomy.

## RESUMO

**Racional:**

Muitas são as injúrias que acometem o fígado e levam a estímulo lesivo. Alguns procedimentos terapêuticos para tratamento dessas lesões dependem da regeneração hepática para aumentar a sua capacidade funcional.

**Objetivo:**

Avaliar o efeito do ácido tranexâmico na regeneração hepática após hepatectomia parcial em ratos.

**Método:**

Foram utilizados 40 ratos (*Rattus norvegicus albinus*, Rodentia mammalia) convencionais da linhagem Wistar-UP. Foram divididos aleatoriamente em dois grupos de 20: grupo controle (CT) e grupo ácido tranexâmico (ATX). Cada um deles foi divido em dois subgrupos para avaliar a regeneração hepática no tempo de 32 h e 7 dias do pós-operatório. A regeneração do órgão foi avaliada quanto ao peso e histologia, sendo esta última por hematoxilina-eosina e antígeno nuclear de proliferação celular.

**Resultados:**

A média dos pesos dos animais dos grupos ATX 7 dias e CT 7 dias no pré-operatório foram de 411,2 g e 432,7 g, respectivamente, e após a regeneração foram de 371,3 g e 392,9 g. As médias das taxas de mitose coradas por HE dos dois grupos em 7 dias foram de 33,7 e 32,6 mitoses, respectivamente, e de 14,5 e 14,9 mitoses para os grupos ATX e CT 32 h. A contagem de células por antígeno nuclear de proliferação celular mostrou valores de 849,7 para o grupo ATX 7 dias e 301,8 para o CT 7 dias; 814,2 para o grupo ATX 32 h e 848,1 para o CT 32 h.

**Conclusão:**

O ácido tranexâmico mostrou-se efetivo na regeneração hepática somente em período mais longo de observação após hepatectomia parcial.

## INTRODUCTION

Hepatic diseases present high morbidity and mortality rates in Brazil; moreover, they are responsible for elevated costs to the Brazilian public health system^3^ ^,^ ^16^. It is known that most of these disorders cause cellular injury; further, when they are not treated adequately, organ failure can be expected, which can lead to imbalance of body homeostasis, deregulation of glycemic level, deficiency in depuration of toxic substances, deficiency of coagulation factors and serum proteins, besides modifications on urea and bile cycle^8^.

Many of used therapeutic procedures, such as live donor transplant and hepatic tumor resection, depend on hepatic regeneration to increase functional capacity of the liver. Given this knowledge, different studies have been performed^6^ ^,^ ^9^ ^,^ ^12^ ^,^ ^17^ ^,^ ^19^.

Following this focus, the role of platelets has been studied. In 2000, the first article about the function of these cells in hepatic regeneration was published. Since then, experimental studies with platelets have been shown that they expressively contribute to the organ regeneration, being their efficiency proportional to their quantity in blood stream^6^ ^,^ ^19^.

The cited papers have identified significant rises at the speed of liver regeneration of guinea pigs with thrombocytosis and abnormal number of platelets, when compared to thrombocytopenic guinea pigs. Moreover, some authors have demonstrated increase of survival of these animals when compared to the last cited group^6^ ^,^ ^19^.

According to the previous hypothesis, it is necessary the verification of drugs that might keep high rates of those elements when cellular injury is observed. Antifibrinolytics, for instance, allow the quantity of platelets to stay static due to the inhibition of fibrinolysis, which prevents or diminishes formation of fibrin degradation products. Furthermore, they decrease the conversion of plasminogen to plasmin, promoting proteolytic activity in the platelet receptors^14^.

The objective of this research was to evaluate the effect of tranexamic acid on hepatic regeneration after partial hepatectomy in rats.

## METHODS

The experiment was conducted at Evangelical Faculty of Paraná, Curitiba, PR, Brazil. The Ethics Committee in Research of the Evangelical Beneficent Society of Curitiba [004117/2013] approved the research project and the ethical principles of handling and experimental animals as defined by the Ethics Committee on Animal Use and Brazilian Society of Science in Animals Lab were respected.

Forty adult male rats (*Rattus norvegicus albinus,* Rodentia mammalia), conventional Wistar lineage were used. They were kept in light/dark cycle of 12/12 h, humidity of 55±10% at room temperature of 21-24ºC, with free access to proper ration for the species and water. The animals were randomly divided into two groups of 20: Control group (CT) and Tranexamic Acid group (TXA). Both were subdivided into two subgroups of 32 h (CT32/TXA32) and 7 days (CT7/TXA7) according to the time of euthanasia after surgery. Initially, the surgical technique consisted in intraperitoneal anesthesia 0.1 ml/100g of ketamine 10% and 0.05 ml/100g of xylazine 2%; epilation; antisepsis; and median laparotomy of approximately 5 cm. The anesthesia was maintained with inhaled isoflurane at a concentration of 1-2.5%.

After the liver was located, it was performed the resection of the round ligament and ligation of the vasculobiliary pedicle of the median lobe with cotton thread 4.0 and later resection. The same procedure was performed in the left lateral lobe, resulting in approximately 70% hepatectomy^7^.

The CT7 and CT32 groups received placebo treatment with infusion of 1 ml/kg of isotonic saline solution 0.9% intraperitoneally after partial hepatectomy. The TXA32 and TXA7 groups received infusion of tranexamic acid (Transamin(r)) at 50 mg/kg into the peritoneal cavity after partial hepatectomy.

The resected lobes were weighed and then laparorrhaphy was made with two suture levels. Postoperative analgesia was performed with tramadol hydrochloride at a dose of 7 mg/kg intramuscularly every 12 h for four days after surgery in CT7 and TXA7 groups, and until the time of euthanasia on CT32 and TXA32 groups.

After the predetermined periods of regeneration, the groups were re-operated to study the regenerated organs. In the immediate postoperative period, all rats were euthanized under overdose inhaled isoflurane. Only CT7 and TXA7 groups were analyzed for estimated regeneration by Kwon formula.

Fixation in buffered formalin to make the slides and study of mitosis with Hematoxylin Eosin (HE) was performed. Slides were also prepared following the method of Tissue Microarray (TMA), characterized by Proliferating Cell Nuclear Antigen (PCNA), for immunohistochemistry quantification of hepatocytes in the replication phase^13^ ^,^ ^15^.

Data were statistically analyzed using the Wilcoxon test, conducted by Action 2.8 program for Microsoft Windows 8, adopting as standard p <0.05

## RESULTS

The weighted averages were 411.2g±10.27 for TXA7 and 432.7g±40.54 for CT7; 391.4g±43.86 for TXA32 and 417g±41.84 for CT32. After the seventh day of surgery, the groups TXA7 and CT7 were weighed again obtaining respectively the averages 371.3g±13.06 and 392.9g±41.28. In Table 1 the weights of each animal can be verified before the partial hepatectomy and after the regeneration.

**TABLE 1:** Weight before the surgery and after seven days of surgery.

	CT32	TXA32	CT7	TXA7
Initial weighing				
Rat 1	390g	460g	390g	418g
Rat 2	415g	300g	379g	390g
Rat 3	340g	330g	400g	420g
Rat 4	390g	370g	430g	410g
Rat 5	440g	411g	470g	415g
Rat 6	415g	403g	372g	415g
Rat 7	505g	415g	447g	410g
Rat 8	440g	410g	446g	420g
Rat 9	440g	410g	503g	420g
Rat 10	395g	405g	400g	394g
Weight after seven days of regeneration				
Rat 1	-	-	372g	380g
Rat 2	-	-	330g	359g
Rat 3	-	-	380g	380g
Rat 4	-	-	410g	370g
Rat 5	-	-	443g	345g
Rat 6	-	-	340g	373g
Rat 7	-	-	420g	385g
Rat 8	-	-	430g	386g
Rat 9	-	-	450g	385g
Rat 10	-	-	354g	360g

The difference between the hepatic regeneration of the groups TXA7 and CT7 when the Kwon's formula was applied was not significant (p=0.91). Analysis by optical microscopy has not demonstrated any change of hepatic cell morphology. The averages number of cells stained by HE were 33.6±12.8 for the TXA7 group, 32.6±10.7 for CT7, 14.5±13.3 for TXA32 and 14.9±20.5 for CT32. The mitotic score differences presented were not significant for both periods of 32 h and seven days (p=0.38 and p=1.0, respectively). The obtained values when the cells were stained with HE are presented in the Table 2.

**TABLE 2:** Number of mitosis stained by HE

	CT32	TXA32	CT7	TXA7
Rat 1	0	4	1	0
Rat 2	62	17	0	0
Rat 3	10	9	0	0
Rat 4	35	46	0	0
Rat 5	0	8	0	0
Rat 6	0	28	0	0
Rat 7	2	12	0	0
Rat 8	28	13	4	1
Rat 9	8	6	0	0
Rat 10	4	2	0	2

By performing immunohistochemistry analysis, the average numbers of cells stained were 849±134 for the TXA7 group, 301.8±241.8 for CT7, 814.2±153.2 for TXA32 and 848.1±52.2 for CT32. The comparison of mitotic values between both groups of 32 h was not statistically significant (p=1.0). However, when both groups of 7 days of regeneration were compared to each other, it was evidenced that the group TXA7 has presented higher mitotic rate (p=0.0002056). The values obtained by the immunohistochemistry stain are presented in the Table 3.

**TABLE 3:** Number of mitosis stained by PCNA

	CT32	TXA32	CT7	TXA7
Rat 1	827	405	90	810
Rat 2	823	853	347	851
Rat 3	948	895	214	868
Rat 4	872	783	854	926
Rat 5	854	947	257	958
Rat 6	769	808	448	620
Rat 7	869	912	40	792
Rat 8	899	888	69	664
Rat 9	792	794	260	959
Rat 10	828	857	439	1049

## DISCUSSION

Understanding the regeneration capacity of the liver and how it will respond to a drug after suffering cellular injury help us to plan better therapies for patients. In the literature, few numbers of similar papers were found; also those studies have not used drugs of the same class, neither the same evaluation period presented in this research. Therefore, the comparison of results is limited.

The determined period of 32 h to evaluate liver response against induced stress was stipulated because at this time after surgery a mitotic peak is observed in the liver. The other determined period of seven days was based on the fact that at this point the regeneration process might be already finished^10^ ^,^ ^18^.

Analysis of liver regeneration by Kwon's formula did not show statistically significant differences. However, this result may be considered doubtful. According to a study published in the Journal of Hepatology^1^, analysis by this method is inefficient, once the body weight may be influenced by different factors, such as inflammatory reaction and deposit of fat and glycogen.

According to a Brazilian research^2^, the evaluation by optical microscopy of growth speed of mitotic figures stained with HE is not a high-accuracy method, since it is impossible to show which cells have entered the cell division cycle, because G1, S and G2 phases of cell life cycle are not identified by optical microscopy. Therefore, the analysis in this study shows to be doubtful too, even that there was equilibrium between the groups. 

On the other hand, PCNA has been shown to be itself an accurate method of measure for cell proliferation analysis, including liver regeneration in rats after partial hepatectomy. PCNA is a useful parameter for analysis of liver regeneration activity in rats after partial hepatectomy^21^. Comparing TXA7 and CT7 groups by immunohistochemistry, it was observed that the group that received tranexamic acid achieved significantly higher regenerative response than the group that did not receive the drug.

In another study^20^, experts have used an immunosuppressant (sirolimus) as drug to induce regeneration. When immunohistochemistry slides with Ki-67 marker were rated, the authors obtained a significant value (p=0.04) comparing the control and experimental groups seven days after partial hepatectomy. Also, a non-significant value in analysis between the 24 h (after surgery) control and experimental groups was found. Based on this, regeneration of liver has shown not be significant in short periods, such as 32 h as demonstrated in this study.

Tacrolimus is another drug that was administered to mice to study hepatic regeneration^4^. The animals were divided into control and experimental groups and were evaluated after 24 h and seven days of hepatectomy. Immunohistochemistry was performed with Ki-67 and PCNA markers. In analysis, the Ki-67 did not show significant differences between the groups. On the other hand, PCNA showed significant differences between the control and study groups after seven days of evaluation, proving the data already submitted. However, in disagreement to the results presented in this study, differences between the 24 h (short time) groups were observed. This discrepancy may be due to different mechanisms of action of the drugs studied.

Because the results obtained in this research have indicated a rise in the mitotic rate after a longer period (seven days) than normal mitotic peak time in cases of liver injury, it can be hypothesized that the drug studied would be delaying this cell activity. However, this assumption is not valid, once the mitotic rate at 32 h of the TXA group should be smaller than the CT group, which was not observed in this study.

One to two hours after partial hepatectomy, it occurs an increase in mitotic growth factors such as HGF (hepatocyte growth factor), which is the most potential mitotic factor, reaching 20 times higher values than those presented before surgery^11^. Three to four days after hepatectomy, the mitotic rate of liver cells is very reduced due to the appearance inhibitors growth factors such as TGF-β1 (transforming growth factor-β1), which is regarded as the signal of the end of the hepatic regeneration^10^. In this period, the liver must have reached almost its original volume. Based on this knowledge, it seems possible that tranexamic acid acts changing the levels of growth factors and inhibition factors^5^.

## CONCLUSION

The experimental model using tranexamic acid has shown to be effective in hepatic regeneration during longer periods of observation after partial hepatectomy.

## References

[1] Assy N, Minuk GY (1997). Liver regeneration methods for monitoring and their
applications. J Hepatol.

[2] Bonhin RG, Carvalho GM, Guimarães AC, Chone CT, Crespo AN, Altemani AMAM, Amstalden EMI (2014). Histologic correlation of expression of Ki-67 in squamous cell
carcinoma of the glottis according to the degree of cell
differentiation. Braz J Otorhinolaryngol.

[3] Carvalho JR, Portugal FB, Flor LS, Campos MR, Schramm JMA (2014). Método para estimação de prevalência de hepatites B e C crônicas e
cirrose hepática - Brasil, 2008. Epidemiol Serv Saude.

[4] G O, Toderke EL, Baretta GAP, Sakamoto DG, Agulham MA, Tambara EM, Maias JEF (2010). Imunossupressão com tacrolimus favorece a regeneração hepática
induzida por hepatectomia extensa em ratos. Arq Bras Cir Dig.

[5] Godoy JL, Matias JEF, Coelho JCU (2006). Regeneração hepática O mito de Prometeu revisado. Jornal Brasileiro de Transplantes.

[6] Gomes HMP, Serigiolle LC, Rodrigues DAB, Lopes CM, Studart SV, Leme PLS (2014). Unfeasible experimental model of normothermic hepatic ischemia and
reperfusion in rats using the Pringle maneuver. Arq Bras Cir Dig.

[7] Higgins G, Anderso G (1931). Experimental pathology of the liver restoration of the liver of the
white rat following partial surgical removal. Arch Pathol Lab Med.

[8] Jesus RP, Waitzberg DL, Campos FG (2000). Regeneração hepática: papel dos fatores de crescimento e
nutrientes. Rev Assoc Med Bras.

[9] Kalil NA, Coral GP, Santos FAI, Gonzalez MC, Neutzling CB (2014). The association between preoperative chemotherapy and the prevalence
of hepatic steatosis in hepatectomy for metastatic colorectal
cancer. Arq Bras Cir Dig.

[10] Kwon AH, Uetsuji S, Yamamura M, Hioki K, Yamamoto M (1990). Effect of administration of fibronectin or aprotinin on liver
regeneration after experimental hepatectomy. Ann Surg.

[11] Lindroos PM, Zarnegar R, Michalopoulos GK (1991). Hepatocyte growth factor (hepatopoietin A) rapidly increases in plasma
before DNA synthesis and liver regeneration stimulated by partial hepatectomy and
carbon tetrachloride administration. Hepatology.

[12] Lopes-Junior AG, Belebecha V, Jacob CE (2014). Hepatectomy a critical analysis on expansion of the
indications. Arq Bras Cir Dig.

[13] Luz L, Sankarankutty A, Passos E, Rizoli S, Fraga GP, Nascimento B (2012). Ácido tranexêmico no tratamento da hemorragia no
trauma. Rev Col Bras Cir.

[14] Matsuo R, Nakamo Y, Ohkohchi N (2011). Platelet administration via the portal vein promotes liver
regenerarion in rats after 70% hepatectomy. Ann Surg.

[15] Michalopoulos GK, DeFrances MC (1997). Liver regeneration. Science.

[16] Morais A, Magno LA, Gomide GPM (2015). Impacto da hepatite C sobre o consumo de recursos e custos de
pacientes com cirrose hepática no SUS. J Bras de Econ Saúde.

[17] Nacif LS, Andraus W, Martino RB, Santos VR, Pinheiro RS, Haddad LBP, D'Albuquerque LC (2014). Adoption of MELD score increases the number of liver
transplant. Arq Bras Cir Dig.

[18] Nocito A, Kononen J, Kallioniemi OP, Sauter G (2001). Tissue Microarrays (TMAS) for high-throughput molecular pathology
research. Int J Cancer.

[19] Silva RR (2010). Regeneração hepática e o papel das plaquetas.

[20] Toderke EL Baretta GAP, Gama O. P. G, Matias J. E. F (2014). Efeito do sirolimo na regeneração hepática induzida por hepatectomia
no rato. Rev Col Bras Cir.

[21] Wolf HK, Michalopoulos GK (1992). Hepatocyte regeneration in acute fulminant and nonfulminant hepatitis
a study of proliferating cell nuclear antigen expression. Hepatology.

